# How methanotrophs respond to pH: A review of ecophysiology

**DOI:** 10.3389/fmicb.2022.1034164

**Published:** 2023-01-06

**Authors:** Xiangwu Yao, Jiaqi Wang, Baolan Hu

**Affiliations:** ^1^Department of Environmental Engineering, Zhejiang University, Hangzhou, China; ^2^Zhejiang Province Key Laboratory for Water Pollution Control and Environmental Safety, Hangzhou, China

**Keywords:** methanotrophs, pH, ecophysiology, ecological distribution, energy trade-off

## Abstract

Varying pH globally affects terrestrial microbial communities and biochemical cycles. Methanotrophs effectively mitigate methane fluxes in terrestrial habitats. Many methanotrophs grow optimally at neutral pH. However, recent discoveries show that methanotrophs grow in strongly acidic and alkaline environments. Here, we summarize the existing knowledge on the ecophysiology of methanotrophs under different pH conditions. The distribution pattern of diverse subgroups is described with respect to their relationship with pH. In addition, their responses to pH stress, consisting of structure–function traits and substrate affinity traits, are reviewed. Furthermore, we propose a putative energy trade-off model aiming at shedding light on the adaptation mechanisms of methanotrophs from a novel perspective. Finally, we take an outlook on methanotrophs' ecophysiology affected by pH, which would offer new insights into the methane cycle and global climate change.

## Highlights

- pH is a significant predictor of microbial communities and biochemical cycles.
- Methanotrophs wildly exist in various acidic and alkaline habitats.
- Various adaption mechanisms such as energy trade-offs have evolved.
- Methanotrophs in acidic and alkaline habitats may become potential methane sinks.

## Methanotrophy in bacteria and archaea

Methane is an important greenhouse gas with a global warming potential of 15–34 times greater than that of carbon dioxide, and its emission contributes to ~20% of global warming (Townsend-Small et al., 2016). Global terrestrial methane emission reaches 370 Tg/year, accounting for 96% of natural methane emissions (Stavert et al., 2020). **Methanotrophs** (see Glossary), utilizing methane as the carbon and energy sources, construct an effective methane filtration system continentally and play significant roles in biogeochemical cycles (Malyan et al., 2016). Methane oxidation is coupled to the reduction of various electron acceptors, including oxygen, sulfate, nitrate, nitrite, manganese (IV), and iron (III) (Hinrichs et al., 1999; Raghoebarsing et al., 2006; Caldwell et al., 2008; Ettwig et al., 2010, 2016; Haroon et al., 2013; Leu et al., 2020). Methanotrophs are usually divided into **aerobic methanotrophs** and **anaerobic methanotrophic (ANME) archaea/bacteria** based on electron acceptor types (Chistoserdova and Kalyuzhnaya, 2018).

**Figure 1:**
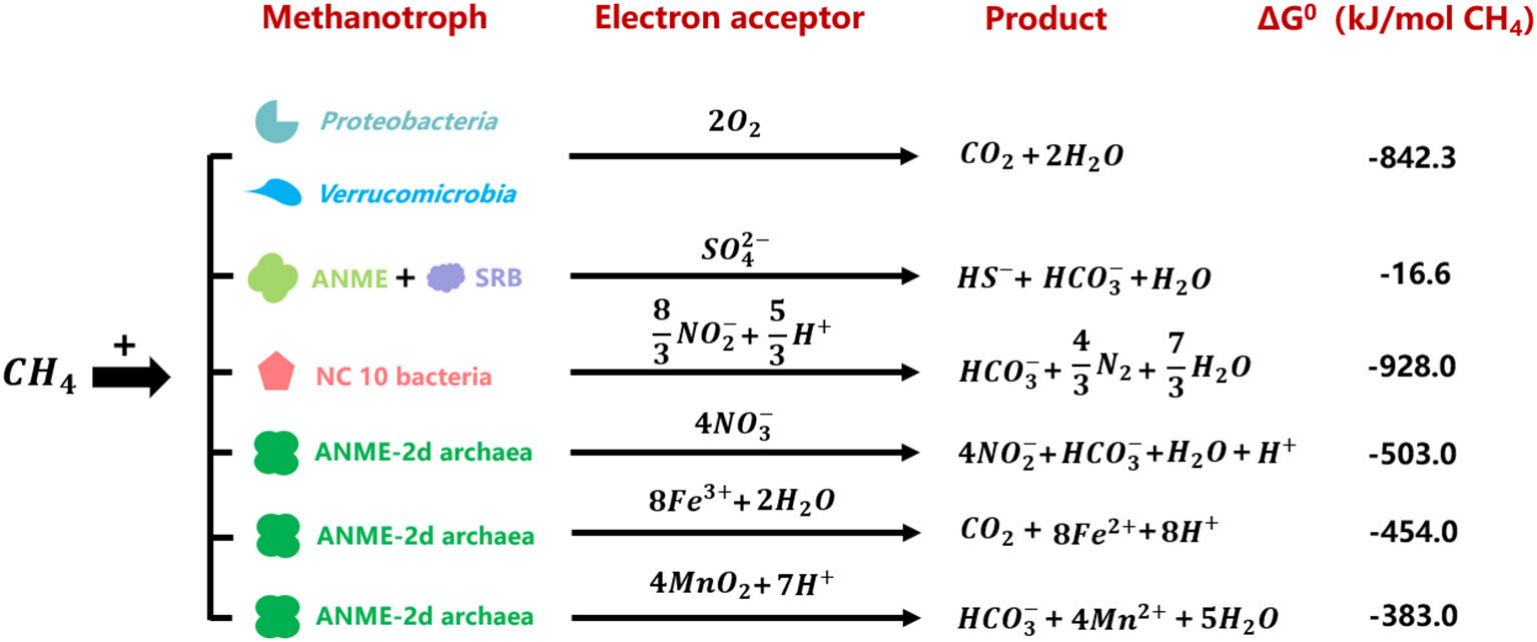
Gibbs free energies of reactions between methane and relevant electron acceptors performed by different methanotrophs (Hinrichs et al., 1999; Raghoebarsing et al., 2006; Caldwell et al., 2008; Ettwig et al., 2010, 2016; Haroon et al., 2013; Leu et al., 2020).

Aerobic methane oxidizers utilize oxygen as the electron acceptor, and they belong to *Proteobacteria* (*Gamma* and *Alphaproteobacteria*) and *Verrucumicrobia*. The former contains gamma-proteobacterial and alpha-proteobacterial methanotrophs (Kalyuzhnaya and Xing, 2018). The latter include the acidophilic genera *Methylacidiphilum* and *Methylacidimicrobium* (Dunfield et al., 2007; Pol et al., 2007; Islam et al., 2008; Schmitz et al., 2021). The anaerobic methane oxidation process refers to methane oxidation with other electron acceptors instead of oxygen. This process is catalyzed by anaerobic methanotrophic (ANME) archaea and bacteria (Hinrichs et al., 1999; Raghoebarsing et al., 2006). However, NC10 phylum bacteria is special for its intracellular oxygen production from nitrite reduction to oxidize methane under anaerobic conditions (Ettwig et al., 2010). To date, five different clusters of ANME archaea have been found: ANME-1, ANME-2a/b, ANME-2c, ANME-2d, and ANME-3 (Baker et al., 2020). **Sulfate-dependent anaerobic methane oxidation (SAMO) process** is catalyzed by ANME-1, ANME-2a/b, ANME-2c, ANME-3 archaea, and their partner sulfate-reducing bacteria (SRB) (Barker and Fritz, 1981; Devol and Ahmed, 1981). **Denitrifying anaerobic methane oxidation (DAMO)** process refers to anaerobic methane oxidation with nitrite or nitrate as the electron acceptors, performed by NC10 phylum bacteria *Candidatus* Methylomirabilis species and ANME-2d archaea *Candidatus* Methanoperedens nitroreducens, respectively (Ettwig et al., 2010; Haroon et al., 2013; Arshad et al., 2015; He et al., 2016; Graf et al., 2018). It was reported that manganese- and iron-dependent anaerobic methane oxidation processes were performed by *Candidatus* Methanoperedens manganireducens/manganicus and *Candidatus* Methanoperedens ferrireducens, which were affiliated with ANME-2d cluster as well (Ettwig et al., 2016; Cai et al., 2018; Leu et al., 2020). Methane oxidation processes coupled to the reduction of different electron acceptors are shown in Figure 1 (Hinrichs et al., 1999; Raghoebarsing et al., 2006; Caldwell et al., 2008; Ettwig et al., 2010, 2016; Haroon et al., 2013; Leu et al., 2020).

## Ecological patterns of methanotrophs driven by pH

pH, which varies on a global scale, largely affects terrestrial microbial communities and biochemical cycles (Fierer, 2017; Tripathi et al., 2018). Delgado-Baquerizo et al. (2018) found that bacterial communities performed pH preferences on a continental scale. Bahram et al. (2018) discovered that global bacterial diversity was regulated by pH and nutrients. Furthermore, shifts induced by global change factors in microbial alpha diversity could be for the main part explained by pH changes in soil (Zhou et al., 2020). Likewise, pH is an essential driving force affecting the ecological niche of methanotrophs. Earlier, the optimal pH for methanotrophs' growth was considered to be 6.6–7.5 (Krulwich et al., 2007). Studies during the last three decades have demonstrated that methanotrophs existed wildly with methane oxidation activity in acidic and alkaline habitats and some of them have been isolated (Trotsenko and Khmelenina, 2002; Semrau et al., 2008; Nguyen et al., 2018; Zhao et al., 2020; Schmitz et al., 2021).

In acidic habitats, such as vast peatlands in the northern hemisphere, multiple proteobacterial methanotrophs have been isolated and characterized since the 1980s (Whittenbury et al., 1970). Various strains affiliated with the genus *Methylosinus, Methylocella, Methylocystis, Methylocapsa, Methylobacter, Methylomonas, Methylovulum*, and *Methyloferula* were considered mildly acidophilic (Heyer and Suckow, 1985; Dedysh et al., 2002, 2015; Dunfield et al., 2010; Iguchi et al., 2010; Svenning et al., 2011; Vorobev et al., 2011; Danilova et al., 2013, 2016; Bowman, 2015; Dedysh and Dunfield, 2016). Verrucomicrobial methanotrophs, different from proteobacterial methanotrophs, were reported to be extremely acidophilic. From 2007 to 2008, three isolations of verrucomicrobial methanotrophs were first obtained from thermal and acidic habitats (Dunfield et al., 2007; Pol et al., 2007; Islam et al., 2008). Subsequently, various novel species were isolated from other acidic environments, which were characterized as metabolically versatile acidophiles (Sharp et al., 2014; van Teeseling et al., 2014; Erikstad et al., 2019; Schmitz et al., 2021). Unlike aerobic methanotrophs, the ecological niche of anaerobic methanotrophs affected by pH was rarely reported. Based on gene sequence analyses, the researchers indicated that both NC10 phylum bacteria and ANME-2d archaea would exist in acidic habitats. Zhu et al. (2015) explored the anaerobic methane oxidation process in Chinese wetland ecosystems and detected the presence of *Candidatus* Methylomirabilis oxyfera (*M. oxyfera*) in the basin sediments with a pH lower than 5. Meng et al. (2016) found the coexistence of anammox and NC10 phylum bacteria in acidic forest soil. Similarly, the presence of *M. oxyfera* was also found in sediment samples from a reservoir in southern China, where the pH was from 5.12 to 5.85 (Long et al., 2017). Compared with the NC10 phylum bacteria, ecological research on ANME-2d archaea is even more lacking. Only a few scholars performed related works. Seo et al. (2014) used the functional gene *mcrA* as a target to detect the presence of *Candidatus* Methanoperedens nitroreducens (*M. nitroreducens*) in acidic paddy soils. Although some anaerobic methanotrophs were detected in various acidic habitats, no systematic research was conducted on the ecological niche driven by pH. At the same time, for lack of isolations, studies on physiology of anaerobic methanotrophs were far from in-depth. The described methanotrophs isolated or detected in acidic habitats are shown in light (mildly acidophilic) and dark (acidophilic) red boxes in Table 1.

**Table 1:** Current described methanotrophs detected or isolated in habitats with different pH.

**Ecotype**	**Phylum**	**Strains**	**pH_optimum_ (and range)**	**Carbon fixation pathway**	**Habitats**	**References**
Acidophilic	*Verrucomicrobia*	*Methylacidiphilum fumariolicum* SolV	2.0 (0.8–6.0)	CBB	Acidic thermal mud pot	Dunfield et al., 2007
		*Methylacidiphilum infernorum* V4	2.0–2.5 (1.0–6.0)		Acidic thermal soil	Pol et al., 2007
		*Methylacidiphilum kamchatkense* Kam1	2.0–2.5 (2.0–5.0)		Acidic thermal spring	Islam et al., 2008
		*Methylacidiphilum* sp. Phi	3.0		Acidic hot spring	Erikstad et al., 2019
		*Methylacidiphilum* sp. Yel	2.8		Acidic hot spring	Erikstad et al., 2019
		*Methylacidimicrobium tartarophylax* 4AC.	1.0–3.0 (0.5–5.5)		Acidic soil	van Teeseling et al., 2014
		*Methylacidimicrobium cyclopophantes* 3B	1.5–3.0 (0.6–5.5)			van Teeseling et al., 2014
		*Methylacidimicrobium fagopyrum* 3C	1.5–3.0 (0.6–5.5)			van Teeseling et al., 2014
		*Methylacidimicrobium* sp. LP2A	3.1 (1.0–5.2)		Acidic mud pool	Sharp et al., 2014
		*Methylacidimicrobium thermophilum* AP8	3.0–5.0 (1.5–5.5)		Acidic geothermal soil	Sharp et al., 2014
Mildly acidophilic	*Proteobacteria*	*Methylocapsa acidiphila* B2	5.0–5.5 (4.2–7.2)	Serine	Acidic peatland	Dedysh et al., 2002
		*Methylocapsa palsarum* NE2	5.2–6.5 (4.1–8.0)			Dedysh et al., 2015
		*Methylocella palustris* K	5.0–5.5 (4.5–7.0)			Dedysh and Dunfield, 2016
		*Methylocella tundrae* T4	5.5–6.0 (4.2–7.5)			Dedysh and Dunfield, 2016
		*Methylocystis bryophila* H2s	6.0–6.5 (4.2–7.6)			Bowman, 2015
		*Methylocystis heyeri* H2	5.8–6.2 (4.4–7.5)			Bowman, 2015
		*Methyloferula stellata* AR4	4.8–5.2 (3.5–7.2)			Vorobev et al., 2011
		*Methylocapsa aurea* KYG	6.0–6.2 (5.2–7.2)		Acidic forest soil	Dunfield et al., 2010
		*Methylocella silvestris* BL2	5.5 (4.5–7.0)			Dedysh and Dunfield, 2016
		*Candidatus* Methylospira mobilis	6.0–6.5 (4.2–6.5)	RuMP	Acidic peatland	Danilova et al., 2016
		*Methylobacter tundripaludum* SV96	(5.5–7.9)			Svenning et al., 2011
		*Methylomonas paludis* MG30	5.8–6.4 (3.8–7.3)			Danilova et al., 2013
		*Methylovulum miyakonense* HT12	(6.0–7.5)		Acidic forest soil	Iguchi et al., 2010
Neutrophilic		Most of methanotrophs	(6.0–8.0)	RuMP/Serine	Soils, lakes, sediments et al.	Whittenbury et al., 1970
Alkaliphilic		*Methylobacter alcaliphilum* 5Z/20Z	9.0–9.5 (7.0–10.5)	RuMP	Soda lake	Khmelenina et al., 1997
		*Methylomicrobium buryatense* strains	8.5–9.5 (6.8-11.0)			Lin et al., 2004
		*Methylomicrobium kenyense* AMO1	9.0–10.0 (9.0–11.0)			Lin et al., 2004
Unknown	NC10 phylum	*Candidatus* Methylomirabilis oxyfera	<5.0^a^	CBB	River	Zhu et al., 2015
			5.3–5.9^a^		Reservoir	Long et al., 2017
			3.8–4.6^a^		Forest soil	Meng et al., 2016
			>9.0^a^		River	Zhu et al., 2015
			9.24^a^		Food waste treatment facility	Xu et al., 2017
	*Euryarchaeota*	*Candidatus* Methanoperedens nitroreducens	5.5–6.4^a^	WL	Paddy soil	Seo et al., 2014
			8.3–10.3^a^		Volcano mud	Ren et al., 2018

CBB, Calvin–Benson–Basham cycle; Serine, serine pathway; RuMP, ribulose monophosphate pathway; WL, Wood-Ljungdahl pathway.

Colors: The described methanotrophs isolated or detected in acidic habitats were shown in boxes with light (mildly acidophilic) and dark (acidophilic) red, those isolated or detected in neutral and alkaline habitats were given in light green (neutrophilic) and light blue (alkaliphilic) boxes respectively, the ecotype of those that remained unknown were shown in light yellow boxes.

^a^Environmental pH range for strains that were not isolated so far.

In alkaline habitats, represented by soda lakes, proteobacterial methanotrophs were isolated and characterized as well. As a unique type of habitat, soda lakes contain a high concentration of carbonate with a pH ranging from 9 to 12. Such harsh condition is unsuitable for microbes due to high salinity, so only a few species affiliated with the genus *Methylomicrobium* and *Methylobacter* were isolated and defined as alkaliphilic methanotrophs (Kalyuzhnaya et al., 2008; Kalyuzhnaya, 2016). From 1995 to 1996, some scholars detected the consumption of methane in soda lakes, indicating the presence of methanotrophs (Sokolov and Trotsenko, 1995; Khmelenina et al., 1996). Khmelenina et al. (1997) isolated two strains of gamma-proteobacterial methanotrophs, *M. alcaliphilum* 5Z and *M. alcaliphilum* 20Z in Tuva soda lake (pH 9.0–9.5). It was the first time to isolate alkalophilic methanotrophs from natural habitats. To explore the activity of methanotrophs in soda lakes, sediments were collected in the Baikal region, and a stable isotope ^13^CH_4_ culture was conducted. DNA-SIP results indicated that gamma-proteobacterial methanotrophs made a major contribution to methane oxidation in such habitats (Lin et al., 2004). Similarly, few research studies focused on anaerobic methane oxidizers in high-pH habitats. The limited studies showed that NC10 phylum bacteria and ANME-2d archaea existed in some alkaline environments as well (Xu et al., 2017; Ren et al., 2018). Xu et al. found NC10 phylum bacterium *M. oxyfera* in food treatment wastewater (pH 9.24). The Pearson correlation analysis showed a significant positive correlation between the pH and NC10 phylum bacterial diversity (Xu et al., 2017). Ren et al. (2018) detected ANME-2d archaea in volcanic mud samples (pH 8.25–10.25) and analyzed the community structure using network methods. Currently described methanotrophs isolated or detected in alkaline habitats were given in light blue boxes of Table 1, and the unknown ecotype is shown in light yellow boxes.

Methane oxidation activities of methanotrophs were conventionally considered to be optimum under neutral conditions (Whittenbury et al., 1970). However, in some acidic or alkaline habitats, the optimum pH values for methane oxidation were in the acidic and alkaline ranges. In some alkaline habitats, it was shown that mud samples from soda lakes exhibited the maximum aerobic methane oxidation rates at pH 8.15–9.40 with a value of 33.2 nmol·ml^−1^·d^−1^ (Khmelenina et al., 2000). The aerobic methane oxidation rate in saline alkaline soils with pH 8.5 was almost as high as that under neutral conditions (pH 6.7) (Serrano-Silva et al., 2014). In some acidic environments, methane oxidation rates reached peaks under low-pH conditions (Brumme and Borken, 1999; Benstead and King, 2001; Levy et al., 2012; Khmelenina et al., 2020). The anaerobic methane oxidation activity in Dianchi Lake was reported significantly and negatively related to pH, and the optimum value was 316.9 nmol·g^−1^·d^−1^ at pH 6.2 (Khmelenina et al., 2020). In acidic forest soil, the maximum aerobic methane oxidation rate (2.88 nmol·g^−1^·d^−1^) was observed at the surface where soil pH was about 4.4 (Benstead and King, 2001). Some acidic forest soil was even regarded as methane sinks with methane uptake rates ranging from 0.02 to 0.49 nmol·m^−2^·s^−1^ in the range of pH 3.85–5.24 (Brumme and Borken, 1999). Similarly, in acidic peatlands, the methane uptake rates reached 100–1400 nmol·m^−2^·s^−1^, where pH values ranged from 4.1 to 6.4 (Levy et al., 2012). Although a high atmosphere methane oxidation rate does not mean that total methane oxidation activity in these habitats is high, a considerable aerobic methane oxidation rate also indicates that methanotrophs living in acidic and alkaline environments might become potential methane sinks contributing directly to methane reduction. To precisely assess the contribution of methane oxidation in acidic and alkaline habitats, more research on the total methane oxidation rate, especially the anaerobic part, should be conducted in the future.

## Physiological adaptions to different pH

**Figure 2:**
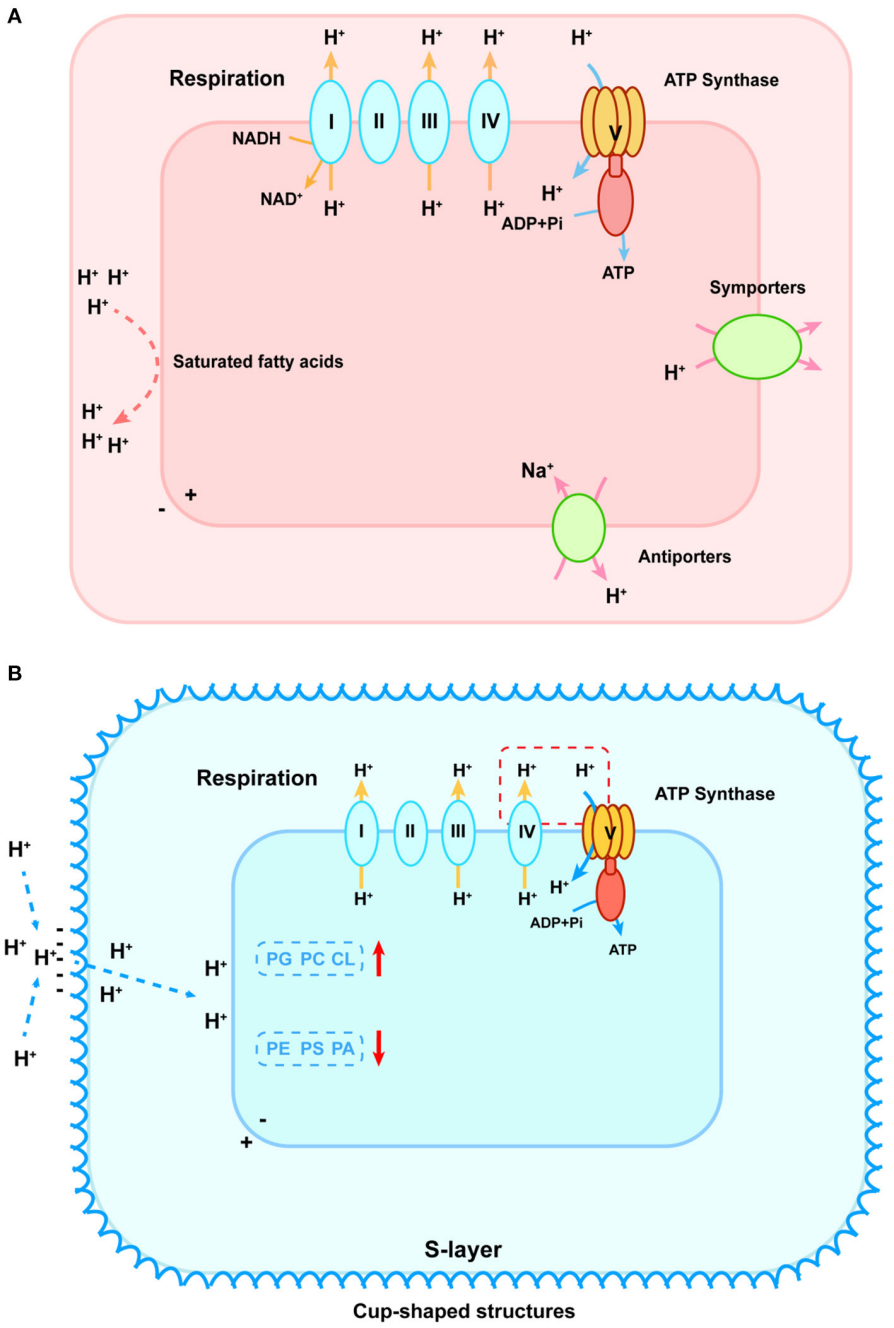
pH homeostasis schematic diagram of acidophilic **(A)** and alkaliphilic **(B)** methanotrophs. **(A)** To stop protons from entering, cytoplasmic membranes with saturated fatty acids are formed, which are shown as red lines on the left. To discharge excess intracellular protons, symporters and antiporters play an important role, shown as green ovals on the right. In the lower-left corner, the blue oval refers to the potassium uptake transporter that helps generate an internal positive membrane potential. The respiration process is shown in the upper part that primary proton pumps (Complexes I, III, and IV) remove protons from the cytoplasm (shown as yellow arrows) that re-enter to generate ATP *via* the F_0_F_1_-ATPase (shown as blue arrows). **(B)** The red dashed box connecting the cytochrome oxidase of the respiratory chain and ATP synthase indicates the existence of incompletely elucidated mechanisms for sequestered proton transfer between the respiratory chain and the ATP synthase in the alkaliphilic methanotrophs. To attract external protons, the S-layer consisting of a monolayer of cup-shaped structures is developed. PG, phosphatidylglycerol; PC, phosphatidylcholine; CL, cardiolipin; PE, phosphatidylethanolamine; PS, phosphatidylserine; PA, phosphatidic acid. The red arrows represent the increase and decrease of these compositions, respectively.

pH makes a difference in microbial metabolism on the cellular level (Nguyen et al., 2018; Daebeler et al., 2020). Microbes synthesize some metabolites with specific structures or compositions to control the proton exchange flux and maintain intracellular pH close to neutral (Semrau et al., 2008; Krulwich et al., 2011). Under low-pH conditions, **acidophilic** microbes employ multiple ways to prevent protons from entering the cytoplasm and discharge excess protons (Slonczewski et al., 2009). The internal positive transmembrane electrical potential helps to maintain a cytoplasmic pH that is only mildly acidic. Owing to the special structure or composition of the cytoplasmic membrane, the influx of protons is blocked as well. The membrane of acidophilic verrucomicrobial methanotrophs was almost made up of saturated fatty acids, whereas membranes of proteobacterial methanotrophs were mainly composed of unsaturated fatty acids (den Camp et al., 2009; Erikstad et al., 2019). It implied that verrucomicrobial methanotrophs required a saturated membrane to minimize proton permeability in an extremely acidic environment (Siliakus et al., 2017). In addition, the relatively greater clusters of a unique gene involved in the cell wall/membrane/envelope biogenesis and secondary symporters/antiporters working to remove excess intracellular protons were found in verrucomicrobial methanotrophs, indicating the structure–function mechanism for coping with acid stress (shown as Figure 2A) (Schmitz et al., 2021). On the contrary, under high-pH conditions, the external positive membrane potential blocks protons off and the second cell wall polymers (SCWPs), such as S-layer protein, are developed by **alkaliphilic** microbes. These components enhance net negative charges on cellular surfaces that increase attraction to external protons (Trotsenko and Khmelenina, 2002; Krulwich et al., 2007). It was reported that alkaliphilic species *Methylomicrobium alcaliphilum* and *Methylomicrobium buryatense* possessed a macromolecular glycoprotein structure (S-layer) wrapped outside the cell. The S-layer consisted of a monolayer of cup-shaped structures (CS) (Kaluzhnaya et al., 2001; Khmelenina et al., 2010). These glycoproteins were composed of a large number of hydrophobic amino acids, lacking sulfur-containing amino acids and exhibiting acidity, which could enhance the net negative charges to attract external protons (Trotsenko and Khmelenina, 2002; Krulwich et al., 2007). Besides, methanotrophs also adapted the composition of the cell membrane to regulate the proton flux. For instance, *Methylomicrobium alcaliphilum* 20Z modified its phospholipid composition based on salinity and pH values (Khmelenina et al., 1997). It increased the relative abundance of phosphatidylglycerol (PG), phosphatidylcholine (PC), and cardiolipin (CL) in response to high pH and decreased the relative abundance of phosphatidylethanolamine (PE), phosphatidylserine (PS), and phosphatidic acid (PA) (Khmelenina et al., 1997). Likewise, an increase in PG relative abundance and a decrease in PA were observed in *Methylomicrobium buryatense* 5G and 7G in response to elevated pH (Kaluzhnaya et al., 2001). Changes in negatively charged phospholipids (PG, CL, and zwitterionic PC) were the most important as they helped accumulate protons on the membrane surface and maintain membrane stability (shown in Figure 2B) (Cullis and Kruijff, 1979).

Methane monooxygenase (MMO) enzyme systems, converting methane to methanol, play a vital role in methanotrophs. It was also reported that MMO could be affected by pH directly or indirectly (Whittington and Lippard, 2001; Ghazouani et al., 2011). Previous structural studies of soluble MMO (sMMO) in *Methylococcus capsulatus* have demonstrated that the hydrogen bonding active site was impacted by pH (Whittington and Lippard, 2001). The results presented that the diiron center in the mixed-valent state at pH values of 8.5 increased liability for ferrous ions in the enzyme. This change altered the surface protein near the catalytic core and resulted in small-molecule accessibility to the active site, which directly affected the activity of sMMO (Whittington and Lippard, 2001). Copper ions are significant for copper-containing particulate MMO (pMMO) regulation and catalysis (Lieberman and Rosenzweig, 2004). pH would indirectly affect the MMO by regulating the Cu uptake process (Ghazouani et al., 2011). Virtually, all methanotrophs, except the *Methylocella* species, can initiate methane utilization through the action of pMMO, while some of them alternately express an sMMO under low copper conditions (Ghazouani et al., 2011; Dedysh and Dunfield, 2016). As methanotrophs expressing pMMO have a high demand for Cu, they develop effective Cu uptake systems (Semrau et al., 2010). One of the Cu acquisition systems is based on the extracellular Cu-binding protein, MopE, or CorA (Helland et al., 2008). Methanobactins (mbs), a class of Cu-binding peptides produced by methanotrophs, were also thought to affect the MMO systems by regulating copper uptake. It was reported that the Cu(I) affinity of mbs was high at pH ≥ 8.0 and one order of magnitude lower at pH 6.0 in *Methylosinus trichosporium* OB3b, indicating that pH might mediate the switchover between sMMO and pMMO by affecting the availability of metal ions (Ghazouani et al., 2011).

**Figure 3:**
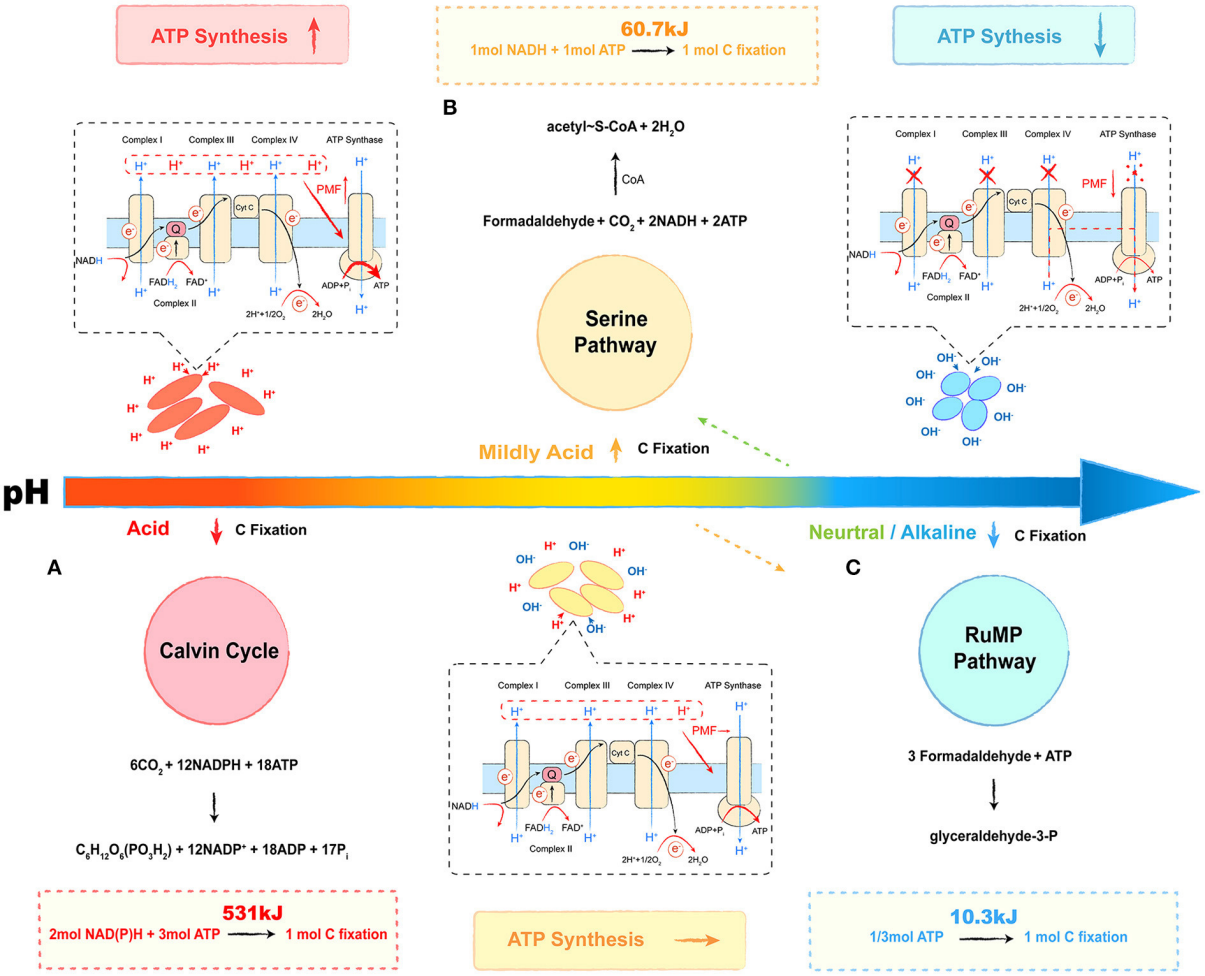
Energy trade-off model of methanotrophs under different pH conditions. The red part refers to the energy trade-off model in acidophilic methanotrophs. The yellow part refers to the energy trade-off model in mildly acidophilic methanotrophs. The blue part refers to the energy trade-off model in alkalophilic methanotrophs. The respiration process is given in the dashed box, and the carbon fixation pathway is shown on the other side. **(A)** Calvin cycle, **(B)** serine pathway, and **(C)** ribulose monophosphate (RuMP) pathway. Most neutrophilic and a few of mildly acidophilic methanotrophs select the RuMP pathway (shown as an orange dotted arrow), and some neutrophilic strains select the serine pathway (shown as green dotted arrow). The overall reactions are shown above or below the cycles, and the energy calculation formulas are followed in the dashed boxes.

Microbial metabolic energy is categorized into three main types: energy produced by catabolism, energy consumed by assimilation metabolism, and heat of reaction (Madigan, 2014). The transfer of electrons between donors and acceptors *via* bio-catalysis is a common energy-producing pathway observed in microbes (Marcus, 2004). During the process, the proton is pumped out to form a proton gradient (ΔpH, chemical potential energy) and a charge gradient (Δψ, electric potential energy). A **proton motive force (PMF)** is formed by the above two potential types of energy, which pushes protons through the membrane back to the cytoplasm and releases energy (Kashket, 1985; Goto et al., 2005; Liu et al., 2008). The ΔpH across the cell is a major contributor to the PMF, suggesting that acidophilic microbes possess the best potential for energy capture, followed by mildly acidophilic or neutrophilic microbes, and alkaliphiles at the bottom (Krulwich et al., 2011; Carere et al., 2021). As for methanotrophs, the oxidation of methane or other electron donors is the main way to capture energy, whereas carbon assimilation is the main way to consume energy (Whittenbury et al., 1970). Although the phylogenetic affiliation plays a key role in selecting the carbon assimilation pathway, the relationship between the pH-ecotype and the carbon fixation pathway of methanotrophs seems intriguing as well. In this review, we provide a putative **energy trade-off** (Ferenci, 2016) model to describe the relationship, which is shown in Figure 3.

The Calvin cycle, the serine pathway, and the ribulose monophosphate (RuMP) pathway are three common ways by which methanotrophs assimilate carbon (Figure 3) (den Camp et al., 2009; Khmelenina et al., 2010; Anvar et al., 2014). The Calvin cycle reactions occur in three basic stages: fixation, reduction, and regeneration. During this process, with CO_2_ as the carbon source, fixing 1 mol C requires 3 mol ATP and 2 mol of reducing power (NADPH), equivalent to 531 kJ energy (Figure 3A). In the serine pathway, the cells use formaldehyde derived from the oxidation of methane as a substrate. The formaldehyde is converted to methylenetetrahydrofolate (CH_2_ =H_4_F) by the tetrahydrofolate (H_4_F) pathway, and CH_2_ =H_4_F enters the serine cycle *via* the demethyltransferase to form acetyl-CoA. In this pathway, fixing 1 mol C requires 1 mol ATP and 1 mol of reducing power (NADH), and the microbial synthesis of the ATP and the reducing power requires about 60.7 kJ of energy, which saves ~90% in energy consumption (Figure 3B). As for the RuMP pathway, formaldehyde is assimilated through two unique reactions: i.e., condensation of formaldehyde and ribulose 5-phosphate to produce hexulose 6-phosphate (Hu6P); ii. isomerization of Hu6P to form fructose 6-phosphate (F6P), which is then converted to pyruvate through the Entner–Doudoroff (EDD) and the Embden–Meyerhof–Parnas (EMP) pathways (Kato et al., 2006; Trotsenko and Murrell, 2008). During this process, for 1 mol C fixation, only 1/3 mol (10.3 kJ energy) of ATP is required, and it only accounts for 2% of the energy in the Calvin cycle (Figure 3C) (Hanson and Hanson, 1996; Semrau et al., 2010). The acidic condition provides a considerable PMF that drives the energy capture process with ATP synthesis. Although acidophilic methanotrophs have to pay a heavy price to survive in such habitats, they still select an energy-consuming pathway, the Calvin cycle, for carbon assimilation, such as verrucomicrobial methanotrophs (Khadem et al., 2012; Sharp et al., 2012). As shown in Table 1, most mildly acidophilic methanotrophs and some neutrophilic strains of *Methylosinus* and *Methylocystis* utilize a relatively energy-efficient pathway, the serine pathway, owing to the decrease of PMF. The lower PMF in neutrophilic and alkalophilic methanotrophs, including a few mildly acidophilic species, requires a more energy-efficient way, the RuMP pathway (Khmelenina et al., 1997; Lin et al., 2004; Iguchi et al., 2010; Svenning et al., 2011; Danilova et al., 2013, 2016). Without sufficient energy, alkalophilic methanotrophs even have to cope with high-pH stress. In this case, selecting the RuMP pathway is undoubtedly a wise strategy for survival (Kato et al., 2006). This putative energy trade-off model might be a miniature of the interaction between microbes and environments with further investigations and verifications. We believe that it will offer novel insights into microbial ecology and biochemical cycles.

## Concluding remarks and future directions

Methanotrophs, an intriguing kind of microbes, play an indispensable role in methane cycles and global climate change. As a significant environmental factor, pH varies significantly on a global scale and is coupled with multiple ecological processes such as nitrogen deposition and precipitation (Galloway et al., 2004; Zhao et al., 2017). It is considered a predictor of microbial communities and biochemical cycles (Fierer and Jackson, 2006; Fierer, 2017). The question that how methanotrophs are affected by pH must deserve to be answered. Based on the current research, this review could answer a part of the question of how methanotrophs respond to pH. However, many challenging issues need to be addressed in this field (see Outstanding questions). How the **methanotrophs' ecophysiology is affected by pH** deserves in-depth studies. In this field, different methanotrophs, especially anaerobic methanotrophs, should be isolated and characterized so that the energy trade-off model can be further verified and physiological mechanisms can be further clarified. Quantitative ecological relationships between pH and methanotrophs need to be established with a complete methane oxidation process, which demands systematic ecological research on both aerobic and anaerobic methanotrophs globally. It is of vital importance to determine the roles that different methanotrophs play in the methane cycle at different pH levels and identify their contributions to the reduction of global methane emissions.

## Outstanding questions

1. What is the ecophysiology of anaerobic methanotrophs affected by pH? Undoubtedly, it requires more ecological investigations and microbial isolations with novel technologies.
2. What is the ecological pattern and methane reduction contribution of methanotrophs under different pH conditions? Is there any methane sink that has been neglected in acidic or alkaline habitats such as peatlands or plateau lakes?
3. How pH impacts the substrate affinity of enzymes in methanotrophs, consisting of the structure–function relationships of sMMO and pMMO and their optimum pH ranges in methanotrophs with various pH-ecotype?
4. Why do acidophilic verrucomicrobial methanotrophs select an energy-consuming pathway rather than an energy-efficient pathway for carbon fixation? Could methanotrophs switch metabolic pathways, such as the carbon assimilation pathway, responding to the change in environmental pH?
5. Verrucomicrobial methanotrophs, as well as proteobacterial methanotrophs, are much more metabolically versatile than previously assumed. Several inorganic gases and other molecules present in acidic geothermal ecosystems can be utilized, such as methane, hydrogen gas, carbon dioxide, ammonium, nitrogen gas, and perhaps also hydrogen sulfide. Could pH matter for metabolic versatility?

## Author contributions

XY: conceptualization and writing—review and editing. JW: writing—review and editing. BH: conceptualization, writing—review and editing, and funding acquisition. All authors contributed to the article and approved the submitted version.

## Glossary

**Methanotrophs:** A type of microbes that utilize methane as carbon and energy sources and play a significant role in natural methane cycles.

**Aerobic methanotrophs:** Bacteria that use molecular oxygen as electron acceptors to activate methane, in a methane monooxygenase reaction.

**Anaerobic methanotrophs (ANME):** Microbes that use other electron acceptors instead of molecular oxygen to oxidize methane, which consists of NC10 phylum bacteria and ANME archaea.

**Sulfate-dependent anaerobic methane oxidation process (SAMO):** A process catalyzed by ANME-1, ANME-2a/b, ANME-2c, ANME-3 archaea, and sulfate-reducing bacteria (SRB) with sulfate as the electron acceptor, oxidizing methane in a reverse methanogenesis pathway.

**Denitrifying anaerobic methane oxidation (DAMO):** Refers to anaerobic methane oxidation with nitrite or nitrate as the electron acceptor, performed by NC10 phylum bacteria and ANME-2d archaea, respectively.

**Acidophilic bacteria:** Extremely acidophilic bacteria grow at external pH <3, whereas the lowest growth pH for mildly acidophiles is in the pH 3–5.5 range.

**Alkaliphilic bacteria:** Extremely alkaliphilic bacteria grow at external pH ≥ 10, whereas moderate alkaliphiles grow in the pH 9–10 range. The pH range for the growth of facultative alkaliphiles extends down to pH 7.0–7.5.

**Energy trade-off:** To spend on one side, energy is forced to economize on the other side. Such negative correlations in microbial energy metabolisms are called energy trade-offs.

**Proton motive force (PMF):** A transmembrane electrochemical gradient across the bacterial cell membrane.

**Methanotrophs' ecophysiology affected by pH:** A systematical ecological and physiological study on methanotrophs coping with pH variation on a global scale. Based on the relationships between pH and climate change, the ecological status and contribution of methanotrophs will be further clarified.
